# Resource Mapping Allocation Scheme in 6G Satellite Twin Network

**DOI:** 10.3390/s22155816

**Published:** 2022-08-04

**Authors:** Zhongliang Deng, Xiaoyi Yu

**Affiliations:** School of Electronic Engineering, Beijing University of Posts and Telecommunications, Beijing 100876, China

**Keywords:** 6G, satellite communication, twin network, integrated network framework, multi-objective optimization

## Abstract

The sixth generation (6G) satellite twin network is an important solution to achieve seamless global coverage of 6G. The deterministic geometric topology and the randomness of the communication behaviors of 6G networks limit the realism and transparency of cross-platform and cross-object communication, twin, and computing co-simulation networks. Meanwhile, the parallel-based serverless architecture has a high redundancy of computational resource allocation. Therefore, for the first time, we present a new hypergraph hierarchical nested kriging model, which provides theoretical analysis and modeling of integrated relationships for communication, twin, and computing. We explore the hierarchical unified characterization method which joins heterogeneous topologies. A basis function matrix for local flexible connectivity of the global network is designed for the connection of huge heterogeneous systems to decouple the resource mapping among heterogeneous networks. To improve the efficiency of resource allocation in communication, twin, and computing integrated network, a multi-constraint multi-objective genetic algorithm (MMGA) based on the common requirements of operations, storage, interaction, and multi-layer optimal solution conflict is proposed for the first time. The effectiveness of the algorithm and architecture is verified through simulation and testing.

## 1. Introduction

The giant satellite Internet constellation has emerged as one of the key visions for 6G communication research. It is significantly different from other 6G communication visions and existing satellite networks in that the giant satellite Internet must be capable of reliable and efficient access on demand in all space and time because of the nonuniform distribution of global users over time and space. It will face unprecedented challenges worldwide, such as on-demand reconfiguration of network node functions under highly dynamic variation in the topology of the giant satellite network, the end-to-end transmission under high-speed links, etc. To address these challenges, research institutions [1,2,3,4] have proposed techniques such as satellite software-defined network (SDN) and large-scale end-to-end slicing, which provide good reference ideas in theoretical exploration. To validate the related techniques, researchers have formed a virtual network based on computational resources by abstracting key functional features of the network and establishing mapping relationships among the features to analyze the network performance under the given network architecture. In fact, they have constructed an integrated model of communication, twin, and computing in a preliminary dimension [5,6,7,8,9,10]. With the research and exploration of the metaverse, it is the key to realizing the intelligent management of 6G networks and studying the innovation of 6G communication protocols, in that a twin network that can simulate and reflect the network operation in high fidelity and detail is constructed.

**Figure 1:**
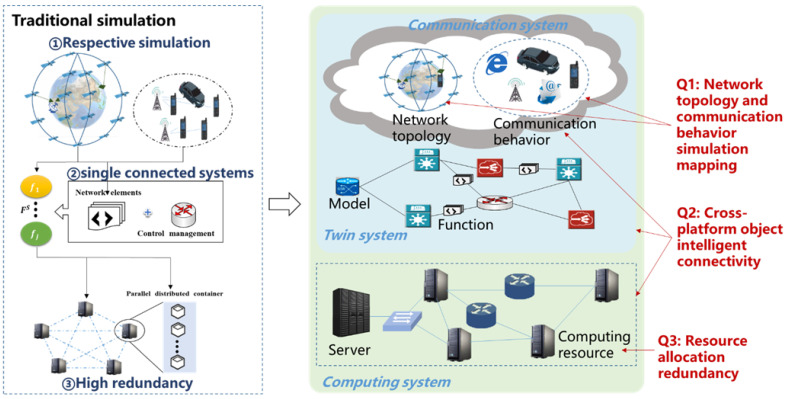
The challenges of mapping and resource allocation in 6G satellite twin network.

As shown in Figure 1, building a 6G satellite twin network faces several difficulties:

1. **Deterministic network geometric topology and fuzzy random communication behavior are difficult to map synergistically**: 6G satellite network simulation needs to realistically portray the network topology and node communication behavior under the rapid change of space and time of massive dynamic network nodes. The network topology is deterministic and discrete. The communication behavior is random and continuous. It is a great challenge for collaborative high-fidelity simulation of network topology and communication behaviors.
2. **The communication network simulation based on computational functions has the problem that cross-platform and cross-object connectivity is isolated**: The communication system, the twin system, and the computing system are completely different research objects in terms of function and logic. The intelligent management of the 6G network needs to realize the penetration of communication, twin, and computing. However, it is difficult to transparently sense the network state.
3. **The redundancy of resource allocation of the communication**–**twin**–**computing integration network is high**: The communication–twin–computing integration network needs to use computing, interaction, storage, and other functions. The implementations of communication, twin, and computing have a large redundancy. There is a major challenge to extract the common functional requirements of communication, twin, and computing in computing, interaction, and storage.

To realize the joint simulation of network geometric topology and communication behavior, the static transmission of network topology position [11], the static transmission of network geometric topology characterization [12,13,14], and synchronous transmission of network topology compression characterization [15] can be generally studied in existing studies. The static transmission of network topology position is to calculate the relative position relationship of the ground nodes and the satellite constellation according to the periodic operational position coordinate information of the satellite constellation. The relative position relationship is used as a premise for the mathematical simulation of random communication behaviors. However, this method cannot transmit the change of satellite position in real time. It is difficult to characterize the real-time network geometric topology range of satellite and ground nodes. The static transmission of network geometric topology characterization is that the connection between the satellite constellation and the ground nodes is used as the input for the random simulation of communication behaviors. Although this method can simulate the discrete connectivity of the network geometric topology of the satellite constellation and the ground nodes, it also does not realize real-time interaction between the network geometric topology and communication behaviors. The synchronous transmission of network topology compression characterization is that the network topology of the satellite constellation and ground nodes is split into a series of event slices over the system cycle, in which each slice describes a fixed network topology over a period of time. This method can synchronously transfer the network topology by dividing the time slices, but the too-small slice division imposes higher requirements on the computer performance and the fidelity of real-time interaction between network geometric topology and communication behaviors.

For the connectivity problem of the communication–twin–computing integration model, object standardization as middleware [16,17], object unification as multi-agent [18,19], service-oriented integration of objects [20,21], microservice-independent object [22,23], and object normalization as function [24] are generally studied. The object of normalization as middleware is to shield the differences between different hardware and software environments and provide a standard, unified transport interface to accomplish transparent access between the communication system, the twin system, and the computing system. However, this approach requires specialized transmission protocols to re-encapsulate the data of systems. The complexity and massive data in the 6G satellite twin network present challenges. Object unification as multi-agent is to view heterogeneous system data as different agents and to unify the data processing commands to act accordingly during data processing. This approach has good scalability, but a large number of agents indicate that the ground nodes can cause a slower processing speed and increase system complexity. The service-oriented integration of objects is to encapsulate the tasks into different services, providing multiple different ways to access the services for heterogeneous systems and reusing the services. However, the service-oriented architecture requires the design of a complex service framework, and it is difficult to be transparently aware of the network state. Microservices-independent object is based on service-oriented with finer granularity and lightweight framework for transmission. However, for the 6G satellite twin network with massive data, too-fine service division leads to too many services and requires efficient computer performance for support. Object normalization as a function normalizes all functions in a heterogeneous system to functions. Functions are scheduled to connect the communication system, the twin system, and the computing system. However, feature extraction and normalization of cross-platform objects are huge challenges.

Facing the problem of high redundancy of resource allocation among heterogeneous systems, to meet the common demands of heterogeneous systems in computing, interaction, and storage, existing studies generally use efficient resource allocation methods such as demand resource adaptive allocation [25,26,27,28], multi-objective dynamic on-demand allocation [29,30,31,32], and artificial intelligence allocation [33,34]. The demand resource adaptive allocation method schedules computational tasks, segments tasks, and manages resources to place tasks by considering parameter size, historical configuration, and running time. Although this method can reduce the transmission time, it is difficult to distinguish the resource for different functions such as computing, interaction, and storage. The multi-objective dynamic on-demand allocation approach can provide a shared resource pool for multiple services simultaneously. Although this method can balance multiple optimization objectives, the multi-objective optimization method only finds the approximate optimal solution. This equilibrium comes at the cost of increased energy consumption. The artificial intelligence allocation method can select the bias of the resource allocation strategy by setting factors to complete the optimization search for the multi-objective problem. However, the multi-level, jointly nested, and diverse computing resources of the communication–twin–computing integration network need to achieve globally optimal cross-system scheduling. Existing artificial intelligence algorithms can only learn and evolve in existing systems. It is difficult to realize the optimization of other systems through its learning.

In order to simulate a 6G satellite twin network, the main contributions of this paper are as follows.

1. For the first time, the integrated relationships among the communication system, the twin system, and the computing system are theoretically analyzed and modeled. For the factor association analysis problem across systems, across dimensions, and across topologies, a hypergraph hierarchical nested kriging model that enables heterogeneous topological connection is introduced for the first time. A hierarchical unified feature description method is realized.
2. In the communication–twin–computing integration network model, the basis function matrix of the local flexible connection of the global network is established. The connection optimization of huge heterogeneous systems is realized, which provides a supportive way to realize the cross-dimensional collaboration of heterogeneous systems.
3. For the first time, a theoretical analysis method that can achieve joint objective optimization of complex heterogeneous systems is proposed. The common requirements of computing, storage, and interaction are also considered to achieve a multi-objective coordinate between function utilization, load balancing, and cost for heterogeneous systems. A cross-platform network simulation architecture with function isolation is proposed for the first time and the effectiveness of the algorithm is verified through experiments.

The rest of this paper is organized as follows. The related work is described in Section 2. The hypergraph hierarchical nested kriging model and the flexible coefficient autoregressive based on the basis function matrix model are given in Section 3. Section 4 analyzes a multi-constraint multi-objective genetic resource allocation optimization scheme. A large-scale network simulation architecture according to the functions is presented in Section 5. The simulation results are illustrated in Section 6. Finally, Section 7 concludes the paper.

## 2. Related Work

With the development of 6G, there are a large number of complex and diverse application scenarios for future 6G mobile communication systems to provide users with an extreme performance experience. For the complex 6G satellite twin network, accurate extrapolation of network performance requires system-level simulation, as well as carefully designed simulation scenarios to better understand the impact of complex relationships between system configuration parameters and corresponding network operations [35]. A semi-physical simulation system is constructed in [36], which uses a server to simulate a satellite and relies on cables and servers to model the channel of the satellite network. The coordinate model to reduce the communication latency of low Earth orbit (LEO) satellites is proposed. However, a large number of servers are required to simulate large-scale constellations under this architecture, which is costly to build. A fifth generation (5G) system-level simulation platform that meets the requirements of IMT-2020 is produced in [1], and its simulation results are verified by the 3rd Generation Partnership Project (3GPP) calibration and published. The modeling of the MAC layer in satellite communications is described in [37,38]. An event-driven satellite performance simulation framework is introduced to simulate the dynamic behavior of the network in the Brazilian region. The user’s operation scenarios are customized in combination with collision detection and different coverage scenarios, but this framework is difficult to scale. The challenges of building a 5G simulation platform based on 4G evaluation methods are described in [39], where a cloud-based framework for a two-level system-level simulation platform framework is proposed and novel techniques in 5G are validated in this platform. A 5G terrestrial system-level simulation platform is introduced in [2], which tests the performance of large-scale multiple-in multiple-out (MIMO) in 5G by varying the number of antenna ports and the number of spatial multiplexing layers in different experimental environments, focusing on the effect of vertical beamforming on full-dimensional MIMO interference, demonstrating that the modular and flexible platform structure is easy to reconfigure and simulate. However, the above simulation platforms are all separate satellite systems or ground systems. The 6G network has flexible and variable protocol stack functions and network architecture, which requires a scalable, multi-compatible, and highly flexible system-level platform for network simulation. In addition, to guarantee transmission stability for the 6G satellite network, low-density parity-check (LDPC) codes and polar codes are selected as the error-correction codes in 5G mobile communications and a lot of standards [40,41,42].

For large-scale simulation systems, some ideas are proposed in current research. A fast, scalable, and parallel simulator to accelerate the simulation of multicore co-processors on a graphics processing unit (GPU) platform is proposed in [43], but this platform depends on hardware conditions. A docker container-based software-as-a-service system is described in [44]. The study demonstrates the feasibility of emulating complete packet-level transmission and protocols on a serverless platform with built-in docker containers and proposes a generic queueing network approach to model the deployment behavior of application-layer software-as-a-service. A serverless cloud computing framework in which applications are modeled as functions of different granularity is proposed in [45]. These functions interact with each other to implement complex task processes. Serverless platforms are mainly used for stateless, data-parallel tasks, and the study verifies the advantages of serverless platforms in solving large-scale optimization problems [46].

For resource allocation of large-scale simulation systems, several studies have been proposed. The tenant demand model and tenant provider model are used to represent the tenant demand, provider demand, and their different attributes and behaviors. A resource deficiency model is designed in [47], and a resource allocation algorithm for resource rejection rate, average resource waiting period, and resource utilization is proposed. The relationship between the resources consumed by task requests and the resources occupied by the system in a discrete computing network is analogized to the relationship between healthy cells and infected cells in epidemiology, and a task-resource model in a discrete computing network based on the classical virus dynamics model is built [48]. I. Al-Azzoni et al. used genetic algorithms and ant colony algorithms to solve the problem of mapping a set of software components to the available computational units in a heterogeneous computing system, but the authors only considered the single-objective problem of component allocation [49]. A model-driven approach was proposed to specify the allocation problem and automatically compute feasible allocations in [50]. The author formulated the allocation problem as a 0–1 integer linear programming; however, the approach can only find feasible allocations (not optimal allocation) and cannot be extended to large-scale scenarios. A platform for implementing architecture optimization algorithms was provided in [51,52,53]. The component deployment problem was modeled as a dual objective optimization problem, but its optimization process stalled after a certain number of iterations. A dynamic allocation algorithm for docker container resources that adapts to application requirements considering node resources, node network consumption, and the energy consumption was proposed in [54]. The resource allocation algorithms when different services are available in docker containers are described in [55,56,57,58]. A genetic algorithm-based resource allocation and task scheduling for minimum completion time and maximum customer satisfaction was proposed in [59] in multi-cloud computing architecture. A hybrid genetic algorithm based on the case base for resource allocation was proposed in [60]. This algorithm finds Pareto solutions in a two-stage algorithm structure by computing crossover operators.

Virtual network mapping is the mapping of a virtual network into a physical network. The deployment of network functions in the virtual network is performed in physical hardware devices through software, which is essentially the allocation of physical resources. The solution to the virtual network mapping problem is mainly to map virtual nodes and virtual links by considering the availability of hardware resources such as computing resources and memory resources of physical nodes in the physical network and the bandwidth resources of physical links, which is the optimal allocation of physical networks for virtual network deployment.

Early virtual network mapping algorithms perform one-to-one mapping for simple networks in a small area, that is, a virtual node in a virtual network request can be mapped to only one physical node. In solving such irreducible mapping problems, the problem is usually modeled as a linear programming problem or a mixed integer programming problem for solution [61,62,62,63]. Although this precise algorithm can traverse each virtual node request, it is only suitable for small virtual network mapping scenarios, and with the expansion of the network scale, the solution time and complexity of the exact algorithm grow exponentially. In addition, some of the early virtual network mapping algorithms [64,65,66,67,68] only consider part of the network requirements, consider the requirements of virtual network nodes in a simple normalized way, or only consider computational resources without considering the impact of storage resources on network mapping. The fidelity and practicality of this type of algorithm are lacking.

With the increase in network scale, the increase in network energy consumption, and the growth of demand for network troubleshooting, virtual network mapping algorithms propose a variety of different solutions for different mapping scenario requirements. For large-scale virtual networks, distributed virtual network mapping algorithms are proposed [69,70,71,72]. The large-scale virtual network is split into multiple small-scale virtual networks, which are deployed as multiple small-scale computational tasks to multiple distributed physical nodes for processing. Finally, the processing information in the distributed physical nodes is collected and aggregated for comprehensive processing of information. An energy-aware virtual network mapping algorithm based on energy consumption was proposed for the increase in network energy consumption [73,74,75,76,77,78]. The number of mapped physical nodes and physical links is taken as the goal of optimization, and the energy consumption of network mapping is controlled by solving for the minimum number of physical nodes and physical links. The survivability virtual network mapping algorithm is proposed for network fault handling requirements [79,80,81,82]. Survivable virtual network mapping algorithms refer to the ability of the network to still provide communication services through the reserved backup resources in case of problems such as natural disasters, network attacks, and equipment failures. The virtual networks and physical networks need to be considered in survivability virtual network mapping algorithms. The main solution considered in survivability virtual network mapping algorithms nowadays is to use the redundancy idea to provide backup resources for the network to resist network failures.

Network mapping for network simulation is studied [83], but communication systems, twin systems, and computing systems are not considered together in a comprehensive manner in the study of network mapping for network simulation. In the large-scale multi-dimensional multi-layer network mapping for network simulation studied in this paper, the mapping problem of the multi-layer heterogeneous network from the communication network to the twin network to the computing network needs to be solved. Among them, the communication network is a large-scale satellite-ground network that consists of nearly 10,000 communication nodes such as ground terminals, high and low orbit satellites, and ground stations distributed around the world. The twin network needs to complete the simulation of nearly 10,000 communication nodes, communication behaviors, and communication processes. The computing network hosts the twin network and provides physical resources such as CPU, memory, bandwidth, and throughput. In the network mapping problem, the traditional virtual network link connection is fixed, but in this paper, the connection between the nodes in the communication network is connected according to the communication behavior and satellite trajectory. The direct connection of different communication nodes changes over time. The network mapping strategy in this paper needs to consider the timeliness of the link connection while satisfying the link resource mapping. In this paper, the communication nodes in the communication network have the attributes of latitude and longitude. The constraint relationship between the geographical locations of the communication nodes needs to be considered when mapping. In view of the different requirements for multi-dimensional multi-layer mapping from the communication network to the twin network to the computing network, the mapping strategies adopted by existing virtualized simulation systems are not applicable to the network mapping in this paper.

In the simulation system studied in this paper, the mapping problem from the communication network to the twin network to the computing network can be summarized as the mapping problem from communication node to twin node to physical node, and communication link to twin link to physical link. After the mapping is completed, the following effects can be achieved: the communication node function can be modeled and reproduced by the twin node, and the physical resources required by the twin node can be carried by the physical node. The communication link function can be modeled and reproduced by the twin link, and the physical resources required and information transmitted by the twin link can be transmitted by the physical link. The amount of resources consumed by the functions carried and transmitted by each physical node and the physical link is basically the same, and the cost of power and energy consumed is minimal.

## 3. System Model of 6G Satellite Twin Network

### 3.1. A Hierarchical Unified Feature Description Method

**Figure 2:**
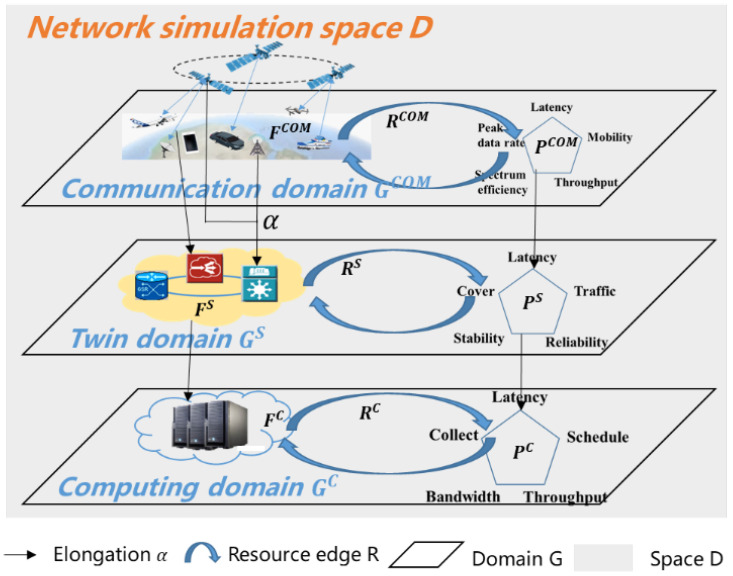
System model.

As shown in Figure 2, the communication–twin–computing integration network is modeled as a space, which consists of three layers: one layer is the communication domain, one layer is the twin domain, and one layer is the computing domain. The communication behaviors in the communication domain are carried by the hardware resources to realize the performance indexes. The communication behaviors and resource load of the communication domain are regarded as the tasks of the twin domain to realize the performance indexes. The twin domain is regarded as the task of the computing domain, which is carried by the parallel distributed docker containers. It is difficult to uniformly model heterogeneous systems that are both independently composed within the system and interconnected, interacting, and nested with each other. We hope that a unified network model can be constructed by combining system characteristics and resource characteristics.

In this paper, as the performance indexes of the communication domain change over time, the twin domain needs to select different functions for simulation implementation. The computation resources required by the communication domain can change accordingly. Therefore, we propose a hypergraph hierarchical nested kriging model based on the characteristics of full-modal and heterogeneous interconnections.

According to complex networks and graph theory, the three-dimensional network can model the content of interest as nodes and edges. The domain is a heuristic abstraction of compositions. The communication domain consists of nodes such as terminals and base stations of real communication networks, hardware resources for building nodes, communication behaviors, and communication performance indexes. The twin domain refers to the simulation of the real communication network functions, resources, environment, behavior, and simulation performance indexes. The computing domain refers to the service resources that constitute the parallel architecture, the computational tasks, and computational performance indexes. In the constructed domain, functional nodes and performance nodes have independent behaviors among themselves, while constituting synergistic behaviors through resource edges. The edges can not only represent connections established based on communication but also can be used to describe diverse associative relationships.

**Definition** **1.** *A hypergraph hierarchical nested kriging space* D={G,P,Bound,α} *: the space D consists of the domain G, the performance index* $P=\left\{p_{1},\cdots ,p_{K}\right\}$ *, the upper and lower limits of the performance index* $Bound=\left(\begin{array}{c} p_{1,max},\cdots ,p_{K,max}\\ p_{1,min},\cdots ,p_{K,min} \end{array}\right)$ *, and the extension factor* α *of the domain G. The range of the space D can be expressed as* $\left(\begin{array}{c} \begin{array}{c} p_{1,min}\leq p_{1}\leq p_{1,max}\\ \vdots \end{array}\\ p_{K,min}\leq p_{K}\leq p_{K,max} \end{array}\right)$ *. The extension factor of the domain G can be expressed as*

(1) $$\alpha \left(G\right)={\left[\alpha_{1}\left(G\right),\cdots ,\alpha_{L}\left(G\right)\right]}^{T}=0.5T\left[\left|p_{d}v_{1}\left(R\right)\right|,\cdots ,\left|p_{d}v_{L}\left(R\right)\right|\right],$$

(2) $$p_{d}=p_{max}-p_{min},$$

(3) $$p_{max}=max\left\{p_{1,max},\cdots ,p_{K,max}\right\},$$

(4) $$p_{min}=max\left\{p_{1,min},\cdots ,p_{K,min}\right\},$$

where *T* is the time interval and $v_{l}\left(R\right)$ is denoted as the orthogonal vector of the vector *R* in the domain (see Appendix B). $p_{d}$ is the maximum difference of the performance index. $p_{max}$ and $p_{min}$ are the maximum and minimum of performance index, respectively.

**Definition** **2.** *The domain G={F,R,P} is defined as a set of hypergraphs. The domain elements $F=\left\{f_{1},\cdots ,f_{J}\right\}$ represents the combinations of the nodes. $R=\left\{r_{1},\cdots ,r_{P}\right\}$ represents the combinations of the edges. $P=\left\{p_{1},\cdots ,p_{Z}\right\}$ represents the performance indexes that divide the domain.*

**Theorem** **1.** *According to Definition 1 and Definition 2, consider a simulation space D={G,P,Bound,α}. The domain G consists of performance indexes $P^{G}=\left\{p_{1}^{G},\cdots p_{J}^{G}\right\}$. The lower bound of each performance index is $l^{G}={\left[l_{1}^{G},\cdots l_{J}^{G}\right]}^{T}$ and the upper bound is $u^{G}={\left[u_{1}^{G},\cdots u_{J}^{G}\right]}^{T}$. Considering a set of node variables $d={\left[d_{1},\cdots d_{N}\right]}^{T}$ in the space D, the node $d_{n}$ exists in the performance index $p_{k}^{d_{n}}$:*

*(1)*  $p_{k}^{d_{n}}\subseteq P$ *and* $p_{k}^{d_{n}}\subseteq P^{G}$*;*
*(2)*  $p_{k}^{d_{n}}\in \left[p_{k,min},p_{k,max}\right]$;
*(3)*  *There are $p_{jmin}^{G}=min\left(\hat{\mu }+Ccvs^{{-1}^{T}}Ccvs\left(l^{G}-1\hat{\mu }\right)\right)$, $p_{jmax}^{G}=max\left(\hat{\mu }+Ccvs^{{-1}^{T}}Ccvs\left(u^{G}-1\hat{\mu }\right)\right)$ (see Appendix A), and $p_{k}^{d_{n}}\in \left[p_{jmin}^{G},p_{jmax}^{G}\right]$.*
  *Then,* $d_{n}\in G=\left(\begin{array}{c} \begin{array}{c} p_{1min}^{G}\leq p_{1}\leq p_{1max}^{G}\\ \vdots \end{array}\\ p_{Jmin}^{G}\leq p_{J}\leq p_{Jmax}^{G} \end{array}\right)$.

According to Definition 1, we build a simulation space D={G,P,Bound,α}. $G=\left\{G^{\mathrm{COM}},G^{S},G^{C}\right\}$ denotes the communication system, the twin system, and the computing system. $P^{G}=\left\{p_{1}^{G},\cdots p_{J}^{G}\right\}$ denotes the performance index in this space: delay, rate, throughput, delay jitter, bandwidth, packet loss rate, etc. The upper and lower bounds of the performance indexes are ${\mathrm{Bound}}^{G}=\left(\begin{array}{c} p_{1\min}^{G},\cdots ,p_{1\max}^{G}\\ p_{\mathrm{Jmin}}^{G},\cdots ,p_{\mathrm{Jmax}}^{G} \end{array}\right)$, and the range of space D can be expressed as $\left(\begin{array}{c} \begin{array}{c} p_{1\min}^{G}\leq p_{1}\leq p_{1\max}^{G}\\ \vdots \end{array}\\ p_{\mathrm{Jmin}}^{G}\leq p_{J}\leq p_{\mathrm{Jmax}}^{G} \end{array}\right)$. There is a set of nodes $d=\left\{d_{1},\cdots d_{N}\right\}$ in the simulation space. 

According to Definition 2, the communication system can be represented as $G^{COM}=\left\{F^{COM},R^{COM},P^{COM}\right\}$. $F^{COM}=\left\{f_{1}^{COM},\cdots ,f_{J}^{COM}\right\}$ denotes the set of functions that make up the communication system. $R^{COM}=\left\{r_{1}^{COM},\cdots ,r_{W}^{COM}\right\}$ denotes the resources required to support the operation of the communication system. $P^{COM}=\left\{p_{1}^{COM},\cdots ,p_{Z}^{COM}\right\}$ denotes the performance indexes of the communication system. According to Theorem 1, when the performance index of the node $p_{d_{n}}^{COM}$ meets the requirements of the communication system, the node $d_{n}$ can be divided into the communication domain.

The twin system can be represented as $G^{S}=\left\{F^{S},R^{S},P^{S}\right\}$. $F^{S}=\left\{f_{1}^{S},\cdots ,f_{J}^{S}\right\}$ denotes the set of functions that make up the twin system. $R^{S}=\left\{r_{1}^{S},\cdots ,r_{W}^{S}\right\}$ denotes the resources required to run each set of functions, $r_{j}^{S}=\left\{\mathrm{cap}f_{j}^{S},\mathrm{mem}f_{j}^{S},\mathrm{ban}f_{j}^{S}\right\}$. $P^{S}=\left\{p_{1}^{S},\cdots ,p_{Z}^{S}\right\}$ denotes the performance index of the twin system. According to Theorem 1, when the performance index of the node $p_{d_{n}}^{S}$ meets the requirements of the communication system, the node $d_{n}$ can be divided into the twin domain. The attributes of resources can be expressed as follows:

**CPU**$capf_{j}^{S}$: indicates the processor resource consumed when processing the function $f_{j}^{S}$.

**Memory consumption**$memf_{j}^{S}$: indicates the memory consumed when processing the function $f_{j}^{S}$.

**Bandwidth**$banf_{j}^{S}$: indicates the data rate when transferring the function $f_{j}^{s}$.

**Function level**$levelf_{j}^{S}$: determines the function level according to the function utilization, which has been defined before using it.

**Function cost**$costf_{j}^{S}$: When the execution time of a function exceeds the specified acceptable time, the cost of processing the function is increased.

The computing system can be represented as $G^{C}=\left\{F^{C},R^{C},P^{C}\right\}$. $F^{C}=\left\{f_{0}^{C},f_{1}^{C},\cdots ,f_{J}^{C}\right\}$ denotes the set of functions running on this computing system. $R^{C}=\left\{\begin{array}{c} s_{11},s_{12},\ldots ,s_{1q},\ldots ,s_{1Q}\\ \ldots \\ s_{P1},s_{P2},\ldots ,s_{Pq},\ldots ,\ldots ,s_{PQ} \end{array}\right\}$ denotes the set of resources available in this computing system. The computing system consists of P servers, each of which is extended with Q docker containers. $P^{C}=\left\{p_{1}^{C},\cdots ,p_{Z}^{C}\right\}$ denotes the performance indexes. According to Theorem 1, when the performance index of the node $p_{d_{n}}^{C}$ meets the requirements of the communication system, the node $d_{n}$ can be divided into the computing domain. $s_{pq}=\left\{\mathrm{cap}s_{pq},\mathrm{mem}s_{pq},\mathrm{ban}s_{pq}\right\}$ denotes the available resource in the docker container q. The attributes of $s_{pq}$ can be expressed as follows:

**CPU**$caps_{pq}$: indicates the available processor resource of the resource $s_{pq}$ in the docker container q.

**Memory**$mems_{pq}$: indicates the available memory of the resource $s_{pq}$ in the docker container q.

**Bandwidth**$bans_{pq}$: indicates the data rate of the resource $s_{pq}$ in the docker container q.

**Resource cost**$costs_{pq}$: indicates the cost of power, software, and equipment spent when processing.

### 3.2. Correlation between Domains within the Network Simulation Space

According to the parameters of the space and domain, each domain in the simulation space is functionally independent of the other. The independence coefficients of the space D can be expressed as

(5) $$Acvs\left(D\right)=\{\begin{array}{c} Ccvs\left(G^{COM},G^{S},G^{C}\right),\alpha \left(G^{COM}\right)+\alpha \left(G^{S}\right)+\alpha \left(G^{C}\right)>1\\ SIM\left(G^{COM},G^{S},G^{C}\right),\;others \end{array},$$

where the $Ccvs(G^{COM},G^{S},G^{C})$ can be expressed separately as

(6) $$\begin{array}{ll} Ccvs(G^{COM},G^{S},G^{C}) & =\sum_{j=1}^{J}\alpha (G^{\mathrm{COM}})\times \log(|r_{j}^{s}|)/\log\{-\frac{\max\{R^{S}\}}{\sum_{p=1}^{p}\sum_{q=1}^{Q}|S_{\mathrm{pq}}|}\}\\ & =\sum_{j=1}^{J}0.5T|p_{d}v_{1}(R^{\mathrm{COM}})|\\ & \times \log(\sqrt{(\mathrm{cap}f_{j}^{S}{)}^{2}+(\mathrm{mem}f_{j}^{S}{)}^{2}+(\mathrm{ban}f_{j}^{S}{)}^{2}})\\ & /\log\{-\frac{\max\{R^{S}\}}{\sum_{p=1}^{p}\sum_{q=1}^{Q}\sqrt{(\mathrm{cap}S_{\mathrm{pq}}{)}^{2}+(\mathrm{mem}S_{\mathrm{pq}}{)}^{2}+(\mathrm{ban}S_{\mathrm{pq}}{)}^{2}}}\}, \end{array}$$

The correlation coefficient between domains is related to the extension factor α of the domain and the resource R of the domain. The correlation coefficient of the domain is obtained based on the CPU $\mathrm{cap}f_{j}$, memory $\mathrm{mem}f_{j}$, bandwidth $\mathrm{ban}f_{j}$, and the extension factor α. The $\mathrm{SIM}\left(G^{COM},G^{S},G^{C}\right)$ can be expressed as

(7) $$\mathrm{SIM}\left(G^{COM},G^{S},G^{C}\right)=\frac{V_{G^{COM}/G^{S}}}{V_{G^{S}/G^{C}}}=\frac{\int_{p_{min}}^{p_{max}}\alpha \left(G^{COM}\right)\cdot \alpha \left(G^{S}\right)\cdot v_{G^{COM}/G^{S}}\left(R\right)dp}{\int_{p_{min}}^{p_{max}}\alpha \left(G^{S}\right)\cdot \alpha \left(G^{C}\right)\cdot v_{G^{S}/G^{C}}\left(R\right)dp},$$

where $v_{G^{COM}/G^{S}}\left(R\right)=max\left\{R^{COM}-sum\left(R^{COM}/R^{S}\right),R^{S}-sum\left(R^{S}/R^{COM}\right)\right\}$ and $v_{G^{S}/G^{C}}\left(R\right)=max\left\{R^{S}-sum\left(R^{S}/R^{C}\right),R^{C}-sum\left(R^{C}/R^{S}\right)\right\}$. We calculate the maximum value of the residual of the ratio of resources between domains and the resources in the domain to obtain the relationship of domains in one space.

### 3.3. Function–Resource Mapping Model

The essence of the communication–twin–computing integration network mapping is to map the communication network topology to the twin network topology and then to the computation network topology. At the same time, the resources are used to abstractly describe the functions. The function F deployed to the resource R can be expressed as H(F)→R in the domain. The twin network is taken as an example for analysis.

The twin function $F^{S}$ deployed to the twin resource $R^{S}$ can be expressed as $H\left(F^{S}\right)\to R^{S}$. We design a flexible coefficient autoregressive model based on the basis function matrix to describe the mapping relationship among the networks.

(8) $$R^{S}\leftarrow H\left(F^{S}\right)=B^{S}\times \left(A\circ F^{S}\right),$$

where $A=\left(a_{1},\cdots a_{J}\right)$ is the matrix containing all linear parameters, $F^{S}$ indicates a function, and $B^{S}=\left(b_{\mathrm{cap}f}^{S},b_{\mathrm{memf}}^{S},b_{\mathrm{banf}}^{S}\right)$ is denoted as the parameter of the Gaussian basis function.

(9) $$A\circ F^{S}=\left(a_{1},\cdots a_{J}\right)\circ \left(f_{1}^{S},\cdots ,f_{J}^{S}\right)=\overset{J}{\underset{j=1}{\sum }}a_{j}\cdot f_{1}^{S}+,\cdots ,+\overset{J}{\underset{j=1}{\sum }}a_{j}\cdot f_{J}^{S},$$

(10) $$b_{f}^{S}=\exp\left(-\frac{\|NUM-c_{f}{\|}^{2}}{2\sigma_{f}^{2}\cdot \sum_{z=1}^{Z}p_{z}^{S}}\right),$$

In Equation (14), NUM is the number of events required by the twin function, $c_{f}$ is denoted as the number of resources required to complete a unit event, and $\sigma_{f}$ is denoted as the variance of resources required to complete a unit event. Then, the parameter of the Gaussian basis function can be expressed as follows:

(11) $$b_{\mathrm{capf}}^{S}=\exp\left(-\frac{\|NUM-c_{capf}{\|}^{2}}{2\sigma_{capf}^{2}\cdot \sum_{z=1}^{Z}p_{z}^{S}}\right),$$

(12) $$b_{\mathrm{memf}}^{S}=\exp\left(-\frac{\|NUM-c_{memf}{\|}^{2}}{2\sigma_{memf}^{2}\cdot \sum_{z=1}^{Z}p_{z}^{S}}\right),$$

(13) $$b_{\mathrm{banf}}^{S}=\exp\left(-\frac{\|NUM-c_{banf}{\|}^{2}}{2\sigma_{banf}^{2}\cdot \sum_{z=1}^{Z}p_{z}^{S}}\right),$$

The mapping relationship of the twin resources and functions can be denoted as

(14) $$\begin{array}{ll} R^{s}=B^{s}\times (A\circ F^{s})= & (\begin{array}{c} b_{\mathrm{capf}}^{S}\\ b_{\mathrm{memf}}^{S}\\ b_{\mathrm{banf}}^{S} \end{array})\times \left(\left(a_{1},\cdots a_{J}\right)\cdot f_{1}^{S},\cdots ,\left(a_{1},\cdots a_{J}\right)\cdot f_{J}^{S}\right)\\ & =\left(\begin{array}{ccc} \overset{J}{\underset{j=1}{\sum }}a_{j}\cdot f_{1}^{S}\cdot b_{\mathrm{capf}}^{S} & \cdots & \overset{J}{\underset{j=1}{\sum }}a_{j}\cdot f_{J}^{S}\cdot b_{\mathrm{capf}}^{S}\\ \overset{J}{\underset{j=1}{\sum }}a_{j}\cdot f_{1}^{S}\cdot b_{\mathrm{memf}}^{S} & \ddots & \overset{J}{\underset{j=1}{\sum }}a_{j}\cdot f_{J}^{S}\cdot b_{\mathrm{memf}}^{S}\\ \overset{J}{\underset{j=1}{\sum }}a_{j}\cdot f_{1}^{S}\cdot b_{\mathrm{banf}}^{S} & \cdots & \overset{J}{\underset{j=1}{\sum }}a_{j}\cdot f_{J}^{S}\cdot b_{\mathrm{banf}}^{S} \end{array}\right)=\left(r_{1}^{S},\cdots ,r_{j}^{S}\right)\\ & =\left(\begin{array}{ccc} \mathrm{cap}f_{1}^{S} & \cdots & \mathrm{cap}f_{j}^{S}\\ \mathrm{mem}f_{1}^{S} & \ddots & \mathrm{mem}f_{j}^{S}\\ banf_{1}^{S} & \cdots & banf_{j}^{S} \end{array}\right), \end{array}$$

where the row is the CPU, memory, and bandwidth required by the function $f_{j}^{S}$, and $a_{j}$ is the mapping factor of the function $f_{j}^{S}$, with values in [0, 1].

## 4. System Optimization Scheme Based on A Multi-Constraint Multi-Objective Genetic Algorithm

In this section, the three optimization objectives of maximizing function utilization, maximizing load balancing, and minimizing total cost are first introduced, and then a multi-objective constrained genetic algorithm (MMGA) is proposed to optimize the system objectives to achieve a dynamic balance of resources and task demands under the constraints of system resources.

### 4.1. Problem Formulation

**Figure 3:**
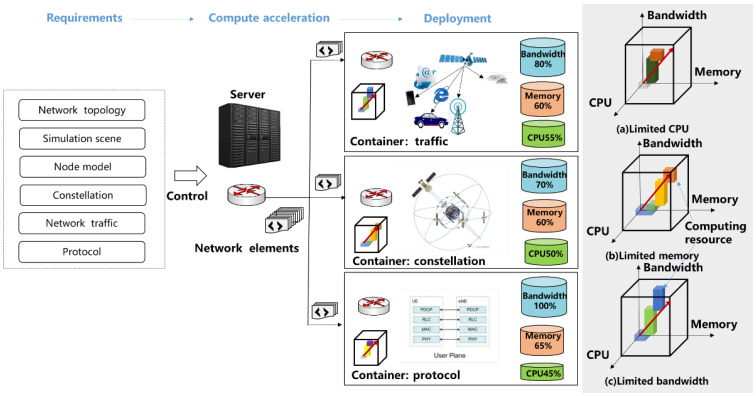
Function–resource three-dimensional packing model.

In the communication–twin–computing integration network, the network function is mapped to the docker containers according to the simulation requirements and the computing resources. The CPU, memory, and bandwidth of the docker containers can be viewed as fixed values before mapping. As shown in Figure 3, the resource in a docker container can be viewed as a box. Thus, we can view the problem of mapping the twin functions to the computing resources as a function–resource three-dimensional packing problem. However, unlike the ordinary three-dimensional packing problem, the function–resource three-dimensional packing problem can only be packed along the diagonal. When the resources of a certain dimension are consumed, it is not possible to continue restarting a new layer of packing. In order to find the optimal solution, we focus on three main objectives: maximizing function utilization, maximizing load balancing, and minimizing the total cost in the 6G satellite twin network.

(1) **Function utilization**

There exists a set of functions that satisfy the event requirements $F^{\prime }=\left\{f_{1},f_{2},\ldots ,f_{j}^{S},\;\ldots f_{J}^{S}\right\}$ in the domain G, where each function corresponds to the set of function utilization values $\left\{futl_{1},futl_{2},\ldots ,futl_{j},\;\ldots futl_{J}\right\}$. Then, the system function utilization can be expressed as

(15) $$\mathrm{utl}F^{\prime }=\overset{J}{\underset{j=1}{\sum }}\frac{1}{J}\cdot \mathrm{level}f_{j}^{S}\cdot futl_{j},$$

Since each QoS has a different effect on the function utilization, each function has a weight of $w_{ij}$. Then, the function utilization $futl_{j}$ can be expressed as

(16) $$futl_{j}=\sum_{i=1}^{3}w_{ij}\cdot \frac{QoS_{i}-QoS_{j}}{QoS_{i}},$$

where $QoS_{j}=\left\{\mathrm{cap}f_{j},\mathrm{mem}f_{j},\mathrm{ban}f_{j},\cos tf_{j}^{S}\right\}$. The $QoS_{i}$ denotes the resources and the cost assigned to the function $f_{j}$, and the $QoS_{j}$ denotes the resources and the cost consumed by the function $f_{j}$.

(2) **Load balancing**

There are two main components in the 6G satellite twin network, namely, distribution balancing of load among individual servers and distribution balancing of load among different docker containers within the same server. The former is used to prevent overloading of individual servers, and the latter is used to prevent overloading of docker containers to ensure efficient utilization of resources. Moreover, when the CPU or memory of a server or docker container is exhausted, the remaining resources, no matter how much is left, can no longer provide services.

The load balancing of all servers can be expressed as

(17) $$loadS=\left(\psi \left(cap\right)+\psi \left(mem\right)+\psi \left(ban\right)\right)\times e^{Acvs\left(D\right)},$$

(18) $$\psi \left(cap\right)=\sqrt{\frac{1}{P}\cdot \overset{P}{\underset{p=1}{\sum }}{\left(\mathrm{cap}F^{\prime }/\overset{Q}{\underset{q=1}{\sum }}\mathrm{cap}s_{pq}-\bar{capF^{\prime }/\mathrm{capS}}\right)}^{2}},$$

(19) $$\psi \left(mem\right)=\sqrt{\frac{1}{P}\cdot \overset{P}{\underset{p=1}{\sum }}{\left(memF^{\prime }/\overset{Q}{\underset{q=1}{\sum }}mems_{pq}-\bar{memF^{\prime }/memS}\right)}^{2}},$$

(20) $$\psi \left(ban\right)=\sqrt{\frac{1}{P}\cdot \overset{P}{\underset{p=1}{\sum }}{\left(banF^{\prime }/\overset{Q}{\underset{q=1}{\sum }}bans_{pq}-\bar{banF^{\prime }/banS}\right)}^{2}},$$

where $\mathrm{cap}F^{\prime }/\sum_{q=1}^{Q}\mathrm{cap}s_{pq}$, $memF^{\prime }/\sum_{q=1}^{Q}mems_{pq}$, $banF^{\prime }/\sum_{q=1}^{Q}bans_{pq}$ denote the utilization of CPU, the utilization of memory, and the utilization of bandwidth in the server p, respectively. $\bar{capF^{\prime }/\mathrm{capS}}$, $\bar{memF^{\prime }/memS}$, $\bar{banF^{\prime }/banS}$ denote the average utilization of CPU, the average utilization of memory, and the average utilization of bandwidth, respectively, which can be obtained by calculating the average resource in all servers and the average requirements of all functions.

Load balancing of the docker containers in server q can be expressed as

(21) $$loadC=\frac{1}{Q}\cdot \sum_{q=1}^{Q}\left(\max\left(\frac{capF^{\prime }}{caps_{pq}},\frac{memF^{\prime }}{mems_{pq}},\frac{banF^{\prime }}{bans_{pq}}\right)-\min\left(\frac{capF^{\prime }}{caps_{pq}},\frac{memF^{\prime }}{mems_{pq}},\frac{banF^{\prime }}{bans_{pq}}\right)\right),$$

where $\frac{capF^{\prime }}{caps_{pq}}$, $\frac{memF^{\prime }}{mems_{pq}}$, $\frac{banF^{\prime }}{bans_{pq}}$ denote the utilization of CPU, the utilization of memory, and the utilization of bandwidth in the docker container p of the server q, respectively

(3) **Total cost**

In this paper, the cost of the 6G satellite twin network is divided into two main parts: one part is the cost of power, software, and equipment, and the other part is the increased cost due to the execution time of the function beyond the specified acceptable time. The total cost required for this network can be expressed as

(22) $$\cos t=\sum_{p=1}^{P}\sum_{q=1}^{Q}\cos ts_{pq}+\sum_{j=1}^{J}\cos tf_{j}^{S},$$

The three optimization objectives in this paper can be formally expressed as follows:

(23) $$\max\left(\mathrm{utl}F^{\prime }\right)=max\sum_{j=1}^{J}\frac{1}{J}\cdot \mathrm{level}f_{j}^{S}\cdot \left(\sum_{i=1}^{4}w_{ij}\cdot \frac{QoS_{i}-QoS_{j}}{QoS_{i}}\right),$$

(24) $$\begin{array}{ll} max\left(loadS+loadC\right) & \\ & =max((\sqrt{\frac{1}{P}\cdot \sum_{p=1}^{P}{\left(\mathrm{cap}F^{\prime }/\sum_{q=1}^{Q}\mathrm{cap}s_{pq}-\bar{capF^{\prime }/\mathrm{capS}}\right)}^{2}}\\ & +\sqrt{\frac{1}{P}\cdot \sum_{p=1}^{P}{\left(memF^{\prime }/\sum_{q=1}^{Q}mems_{pq}-\bar{memF^{\prime }/memS}\right)}^{2}}\\ & +\sqrt{\frac{1}{P}\cdot \sum_{p=1}^{P}{\left(banF^{\prime }/\sum_{q=1}^{Q}bans_{pq}-\bar{banF^{\prime }/banS}\right)}^{2}})\times e^{Acvs\left(D\right)}\\ & +\frac{1}{Q}\cdot \overset{Q}{\underset{q=1}{\sum }}(\max\left(\frac{capF^{\prime }}{caps_{pq}},\frac{memF^{\prime }}{mems_{pq}},\frac{banF^{\prime }}{bans_{pq}}\right)\\ & -\min\left(\frac{capF^{\prime }}{caps_{pq}},\frac{memF^{\prime }}{mems_{pq}},\frac{banF^{\prime }}{bans_{pq}}\right))), \end{array}$$

(25) $$\min\left(\cos t\right)=\min\left(\sum_{p=1}^{P}\sum_{q=1}^{Q}\cos ts_{pq}+\sum_{j=1}^{J}\cos tf_{j}\right),$$

s.t.

(26) $$\mathrm{cap}S\geq \mathrm{cap}F^{\prime },$$

(27) $$\mathrm{mem}S\geq \mathrm{mem}F^{\prime },$$

(28) $$\mathrm{ban}S\geq \mathrm{ban}F^{\prime },$$

### 4.2. The Multi-Constraint Multi-Objective Genetic Algorithm

To efficiently allocate resources in the 6G satellite twin network, a multi-objective optimization model that considers function utilization, load balancing, and the cost is developed. Multi-objective optimization problems are basically discussed based on Pareto optimal solutions, where multiple-objective components are composed into a vector and the minimization of the vector is solved. Based on the above idea, the objective problem is modeled as follows:

(29) $$\begin{array}{ll} \min f\left(F,S\right) & =\min\left(f_{1},f_{2},f_{3}\right)\\ & =\min\left(\left(1-\max\left(\mathrm{utl}F^{\prime }\right)\right),\;\min\left(1-max\left(loadS+loadC\right)\right),\left(\min\left(\cos t\right)\right)\right), \end{array}$$

In contrast to single-objective optimization problems, multi-objective optimization problems have difficulty finding a solution that makes all the objective functions optimal at the same time due to the conflict between the objectives. Therefore, multi-objective optimization problems usually have a solution set. Each solution in the solution set is the non-dominated solution of all the objective functions. It is impossible to compare the advantages and disadvantages of each solution for the whole objective function.

A multi-constraint multi-objective genetic algorithm is proposed to find a non-dominated solution to the problem. We use a non-dominated hierarchical approach, which allows more suitable non-dominated solutions in each layer to have a greater chance of being inherited to the next selection during the solution process. At the same time, the fitness sharing strategy is used to make the non-dominated solutions in each layer uniformly distributed to maintain population diversity, avoid overpopulation of super individuals, and prevent premature convergence.

In the ath-level domination layer there exists $b_{a}$, the non-dominated solution; the distance between the two solutions, $\left(F_{m}^{\prime },{\;S}_{m}\right)$ and $\left(F_{n}^{\prime },S_{n}\right)$, which represents the deployment of the function in the resource, can be expressed as

(30) $$d\left(m,n\right)=\sqrt{{\left(\frac{\left(F_{m}^{\prime },S_{m}\right)-\left(F_{n}^{\prime },S_{n}\right)}{{\left(F_{m}^{\prime },S_{m}\right)}^{u}-{\left(F_{m}^{\prime },S_{m}\right)}^{d}}\right)}^{2}},m,n=1,2,\ldots ,{\;b}_{a},$$

where ${\left(F_{m}^{\prime },S_{m}\right)}^{u}$ and ${\left(F_{m}^{\prime },S_{m}\right)}^{d}$ are the upper limit and the lower limit of the $\left(F_{m}^{\prime },S_{m}\right)$, respectively.

The shared function SH represents the relationship between the non-dominated solution $\left(F_{m}^{\prime },S_{m}\right)$ and other non-dominated solutions within this layer, denoted as

(31) $$\mathrm{SH}\left(d\left(m,n\right)\right)=\{\begin{array}{c} 1-{\left(\frac{d\left(m,n\right)}{r_{ave}}\right)}^{\beta }d\left(m,n\right)<r_{ave}\\ 0\;\;\;\;\;\;\;other \end{array},$$

where the $r_{ave}$ is the average of the non-dominated solution $\left(F_{m}^{\prime },S_{m}\right)$ and the radii of other non-dominated solutions within this layer, and β is a constant.

The number of non-dominated solutions in this dominated layer can be expressed as

(32) $$c_{m}=\sum_{m=1}^{b_{a}}s\left(d\left(m,n\right)\right),$$

The shared fitness of the non-dominated solution $\left(F_{m}^{\prime },S_{m}\right)$ can be expressed as

(33) $$h_{m}=\frac{s\left(d\left(m,n\right)\right)}{c_{m}},$$

A selection strategy based on the k-means algorithm and proportional distribution is used in each level of the dominance layers to select the optimal solution from the elite pool so that individuals are distributed along the Pareto front. The results in this dominance layer can be expressed as

(34) $$KNum_{m}=sgn\left(h_{m}-\sqrt{\sum_{k=1}^{3}{\left[\frac{f_{k}\left(\left(F_{m}^{\prime },S_{m}\right)\right)-f_{k}\left(\left(F_{n}^{\prime },S_{n}\right)\right)}{f_{k}^{max}\left(F,S\right)-f_{k}^{min}\left(F,S\right)}\right]}^{2}}\right),$$

where $f_{k}\left(\left(F_{m}^{\prime },S_{m}\right)\right)$ is the solution of the kth objective function. $f_{k}^{max}\left(F,S\right)$ and $f_{k}^{min}\left(F,S\right)$ are the maximum and minimum values of the kth objective function, respectively. sgn is the sign function, which means that when the unit ball contains more dominated solutions, $KNum_{m}$ will be larger.

## 5. Serverless-Based Decentralized Simulation Development Model

In this section, we describe the design of the three-dimensional hierarchical simulation framework and the decentralized adaptive development model. The component architecture and application process of the 6G satellite twin network are introduced.

### 5.1. The Three-Dimensional Hierarchical Simulation Framework

**Figure 4:**
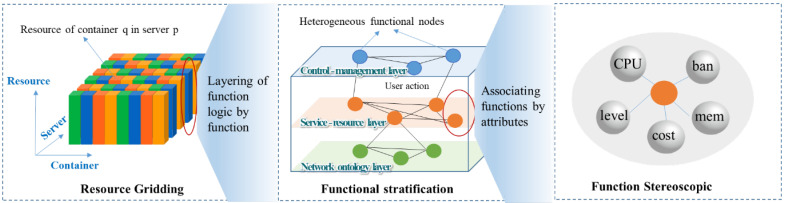
The three-dimensional hierarchical simulation framework.

The 6G satellite twin network includes simulation network initialization, simulation task segmentation, simulation task assignment mapping, function combination, resource balance coordination, and simulation task process. In this paper, a three-dimensional hierarchical simulation framework is proposed, as shown in Figure 4. In this framework, CPU, memory, and bandwidth are collectively referred to as resources. The resources are gridded according to the distribution of the docker containers of the servers and modeled as $s_{pq}=\left\{\mathrm{cap}s_{pq},\mathrm{mem}s_{pq},\mathrm{ban}s_{pq}\right\}$. All tasks of the docker containers are performed by the function $F_{pq}^{C}=\left\{f_{0}^{C},f_{1}^{C},\cdots ,f_{J}^{C}\right\}$. The resources support the functions to be executed. The docker containers interact according to the contact of the function.

In the 6G satellite twin network, we decouple resources, transport, control, and task. The resources of each docker container are logically layered according to function into the control management layer, service resource layer, and network ontology layer.

**Control-management layer**: This layer includes the functions for network architecture. The simulation network initialization, the connection between different docker containers, and the transmission of communication data and control information between docker containers are carried by this layer.

**Service-resource layer**: This layer consists of task requirement discovery functions and resource orchestration mapping functions. The hardware resources are allocated to the functions. This layer completes the load balancing between different servers and docker containers.

**Network ontology layer:** This layer includes the ontology functions of the 6G satellite twin network. The network topology, simulation scenarios, node models, constellations, network traffic, and protocols are simulated.

A 6G satellite twin network is decomposed into multiple sub-simulation task sets distributed into different docker containers. Different simulation tasks are run in different docker containers. The differences in the functions that make up the simulation tasks lead to task differences between the docker containers. The consistency of the functions in the docker containers varies.

### 5.2. The Decentralized Adaptive Development Model

**Figure 5:**
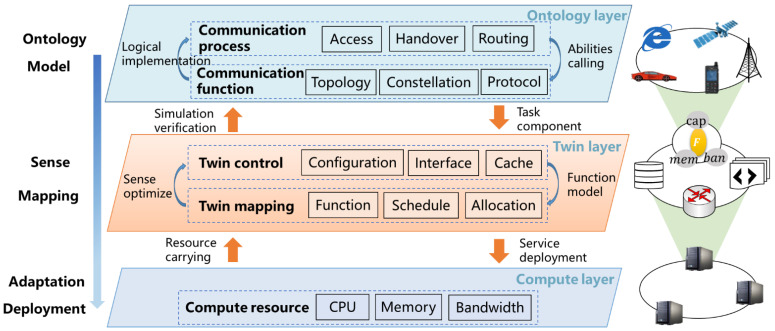
The decentralized adaptive development model.

The development of the 6G satellite twin network needs to consider the support and connection between the communication system, the twin system, and the computing system. The global control and management of the communication–twin–computing integrated network are achieved through the grid-based management of resources and the logical hierarchy of functions. Thus, various requirements in the integrated network can be further developed and described by abstracting them into virtualized functions. Based on the analysis, we propose a new decentralized adaptive development model, as shown in Figure 5.

As shown in Figure 5, the proposed model includes an ontology layer, a twin layer, and a computing layer. The ontology of the simulation can be abstracted into behaviors and functions. The ontology layer of the 6G satellite twin network in this paper can be abstracted into communication behaviors such as access, handover, and routing. The communication behaviors are implemented logically through communication functions such as topology, constellation, and protocol to simulate the communication capability. The twin layer is the control and mapping of the twin network. The control and management functions such as configuration, interface, and cache in the twin network are represented by functions. The optimal allocation of functions and resources is calculated according to the resource requirements of all functions to achieve the optimal utilization of resources in the 6G satellite twin network, that is, on the basis that the resources consumed by the functions do not exceed the resource capacity provided by all servers, as many simulation tasks as possible are completed, and the tasks allocated in each docker container are balanced more effectively to achieve the optimal simulation time or optimal utilization of resources. In the computing layer, functions are deployed according to the optimal allocation of resources and functions to meet the execution of simulation tasks.

The development idea of the model is to establish the mapping relationship among functions and resources by sensing the correlation among ontology function models. Based on the mapping relationships, the function–resource adaptation relationships are calculated and the functions are deployed into the corresponding docker containers.

## 6. Results and Discussions

**Figure 6:**
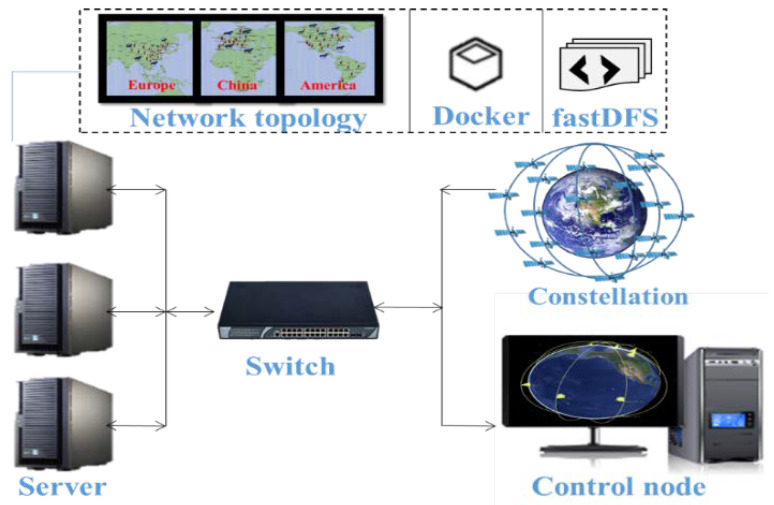
Simulation platform.

As shown in Figure 6, in order to test the performance of the 6G satellite twin network, we built a 6G satellite twin network simulation platform consisting of servers, switches, control nodes, constellation, and protocol. As shown in Table 1, we used the servers with a core i7 4.6 GHz CPU, 8 GB RAM, and 512 GB SSD. We considered 1000 users with a data rate of 100 kbps, mainly distributed in China, Europe, and America. A Walker constellation of 64 satellites deploying 1200 km altitude was built, distributed in eight planes on average. RRC/SDAP/PDCP/RLC/MAC/PHY protocol layers are running in the satellites and users. A parallel computing platform with serverless architecture is used for simulation.

**Figure 7:**
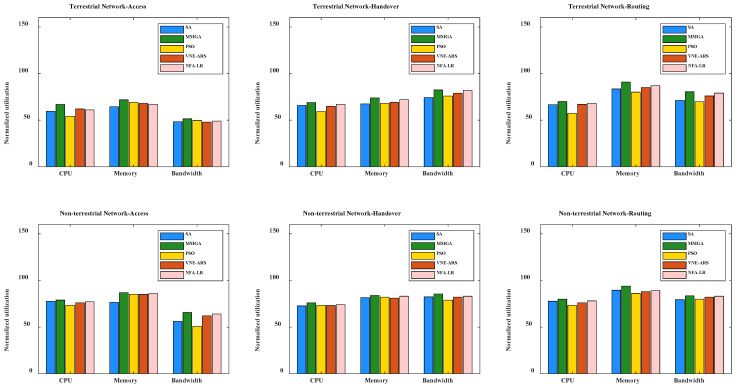
The CPU, memory, and bandwidth normalized utilization of different algorithms under access, handover, and routing behaviors in the terrestrial network and the non-terrestrial network.

In this paper, we chose two traditional methods for solving optimization problems, namely, the simulation annealing algorithm (SA) and the particle swarm optimization algorithm (PSO), to compare with the MMGA proposed in this paper. The SA [84,85,86] and the PSO [87,88,89] are also frequently used in network mapping problems for objective optimization. In addition, we chose the virtual network mapping algorithm based on hierarchical relationship (NFA-LR) [90] and virtual network embedding based on attributes and resources for single-stage (VNE-ARS) [91] for comparison with the MMGA. We compare the normalized utilization of terrestrial networks and non-terrestrial networks in terms of access, handover, and routing behaviors. The normalized utilization of CPU, memory, and bandwidth with different algorithms is shown in Figure 7. It can be seen from Figure 7 that the MMGA, which is used to find the optimal utilization, can effectively improve resource utilization compared with other algorithms. At the same time, the normalized utilization of non-terrestrial networks is higher than that of terrestrial networks in terms of CPU, memory, and bandwidth. This is because unlike the terrestrial network, constellations are added to the non-terrestrial network. Satellites in a non-terrestrial network can perform different functions. When the satellite has the transparent forwarding function, the satellite is responsible for data forwarding between the terminal and the base station, which requires less processing capability for the satellite. When the satellite is used as the central unit processing function of the onboard base station, the satellite is mainly responsible for the access function of the user terminal, but it also needs to forward the access information to the ground base station to complete the data return. When a satellite acts as an onboard base station with all base station functions, the satellite is responsible for user terminal access and user data backhaul services. In the latter two functions, the processing capability of the satellite is required to be higher. In addition, the bandwidth utilization of the handover behaviors is significantly higher than that of the access and handover behaviors, which is due to that more information is exchanged between different network nodes in the handover behavior than in the access and routing behaviors. The memory utilization in the routing scenario is higher than that in the access and handover behaviors due to the need to store node states for routing.

**Figure 8:**
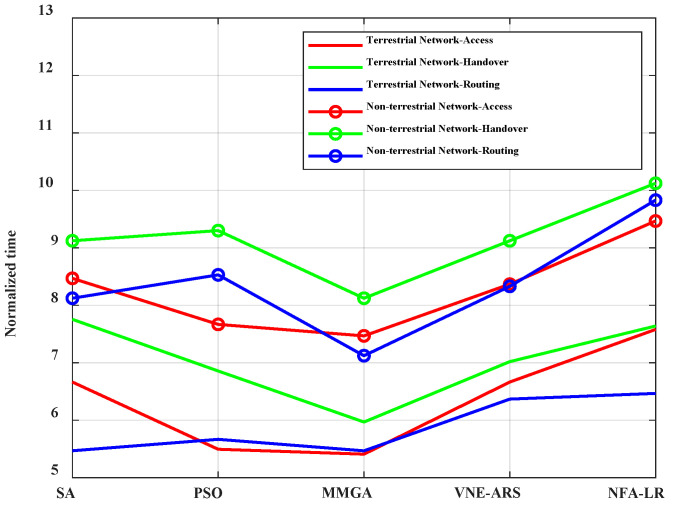
Normalization time consumed for different algorithms.

The time consumed by using SA, PSO, MMGA, VNE-ARS, and NFA-LR algorithms in different scenarios is shown in Figure 8. The time running the non-terrestrial network is significantly higher than that running the terrestrial network. The MMGA algorithm proposed in this paper takes less time than the rest of the algorithms. This is because the time is taken as cost as the optimization objective in this paper. The optimal resource allocation scheme is obtained in the least amount of time.

**Figure 9:**
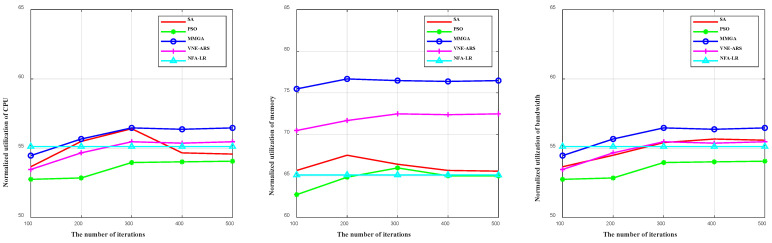
The CPU, memory, and bandwidth normalized utilization of different algorithms under the different number of iterations.

In Figure 9, we verify the effect of different algorithms on the normalized utilization of CPU, memory, and bandwidth for the different number of iterations. We can observe that as the number of iterations increases, the CPU normalized utilization of the PSO, the VNE-ARS, and the MMGA increases to a smooth maximum. However, as the number of iterations increases, the CPU normalized utilization of the SA increases and then decreases. At the same time, as the number of iterations increases, the memory normalized utilization of the SA and the PSO increases and then decreases, and the memory normalized utilization of the VNE-ARS and the MMGA increases to a smooth maximum. In addition, the bandwidth normalized utilization of the SA, the PSO, the VNE-ARS, and the MMGA increases to a smooth maximum. This is due to the overconvergence of the SA as the number of iterations increases. Since we assign the maximum weight to the optimal solution of each layer in the solution of the MMGA, it has a better chance of being inherited to the next layer, avoiding the overproduction of super-individuals and preventing premature convergence. Meanwhile, in the MMGA, the solutions with the lowest 10% weight values are eliminated at each iteration. In this way, as the number of iterations increases, the number of individuals to be searched for the best gradually decreases and the resources consumed do not show a linear increase. However, due to the increase in the number of computations, resources are still consumed. In addition, no iteration is performed in the NFA-LR, so the normalized utilization of CPU, memory, and bandwidth does not change as the number of iterations increases.

**Figure 10:**
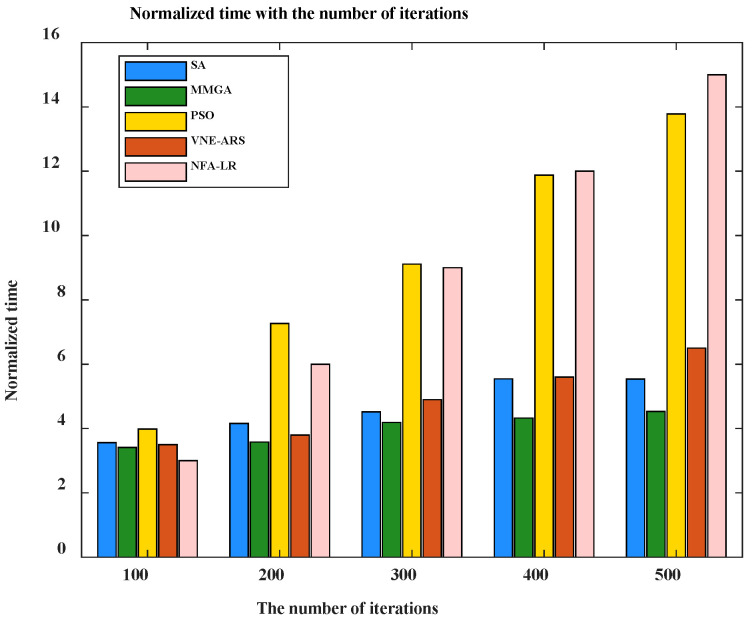
Normalization time consumed for different numbers of iterations.

From Figure 10, we can observe that the time consumed by different algorithms varies as the number of iterations increases. This is due to the increase in the number of iterations leading to an increase in the optimal result of each algorithm. In addition, the SA, the PSO, the VNE-ARS, and the NFA-LR are highly affected by the iteration time. The MMGA is less affected by the iteration time. This is because the cost of consumption is used as a limiting condition for finding the optimal result in the MMGA. Minimization time is considered in the search for the optimal allocation result.

**Figure 11:**
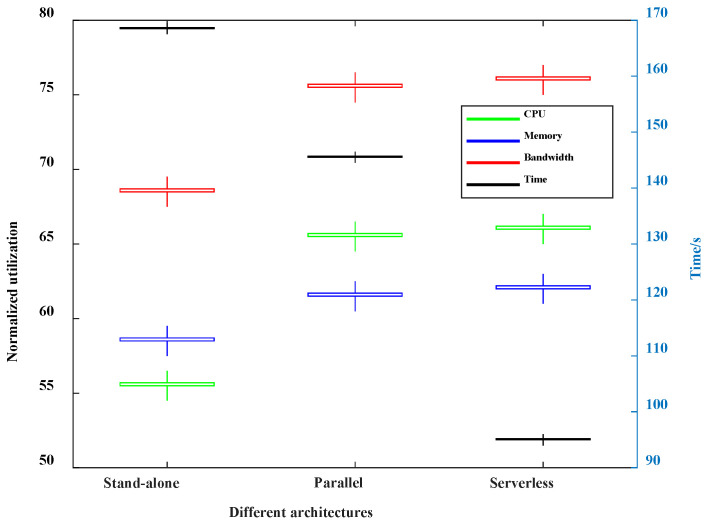
Computing performance under different architectures.

In this paper, we simulate the communication system using standalone architecture, parallel architecture, and serverless architecture. The performance is shown in Figure 11. As shown in Figure 11, the normalized utilization using standalone architecture is much smaller than that using the parallel and serverless architectures among CPU, memory, and bandwidth. The normalized utilization of the parallel architecture and serverless architecture is the same, because the servers of these architectures can process the tasks synchronously, but the tasks of standalone architecture can only be processed in one server at the same time. In addition, the processing time of the standalone architecture is the largest. The transmission of control information increases the processing time due to a master control node needing to control all servers in the parallel architecture. The control information transmission time of serverless architecture is less than that of parallel architecture due to the means of decentralized control, so the serverless architecture consumes the least simulation time.

## 7. Conclusions

In this paper, we discuss the theoretical analysis and modeling of the 6G satellite twin network, in which the key issues of the communication–twin–computing integration network model are investigated. A hypergraph hierarchical nested kriging model and a hierarchical unified feature description method are proposed to describe the communication network and twin network and computing network. Meanwhile, the basis function matrix of the local flexible connection of the global network is introduced to map the functions to the resource. In addition, the optimal mapping problem is described as a three-dimensional packing problem, and the multi-constraint multi-objective genetic algorithm is proposed to find the optimal computational resource allocation. Finally, the three-dimensional hierarchical simulation framework and the decentralized adaptive development model are proposed.

## Figures and Tables

**Table 1:** Simulation parameters configuration.

Classification	Configuration	Parameter
Hardware	CPU	i7 4.6 GHz
	Memory	8 GB
	SSD	512 GB
Simulation	Constellation	Walker
	Satellite altitude	1200 km
	Number of satellites	64
	Number of satellite planes	8
	Distribution of users	Europe, China, America Poisson distribution
	Number of users	1000
	Protocol	RRC/SDAP/PDCP/RLC/MAC
	Code	LDPC/Polar
	Modulation	BPSK/QPSK

## Data Availability

Not applicable.

## Appendix A

There is a set of performance lower bounds $l^{G}={\left[l_{1}^{G},\cdots l_{J}^{G}\right]}^{T}$ for the domain G with the mean μ and the variance $\sigma^{2}$. The correlation of the domain G can be expressed as

(A1) $$\begin{array}{ll} Ccvs(G^{1},G^{2}) & =\sum_{j=1}^{J}\alpha (G^{1})\times \log\left(\left|r_{j}^{1}\right|\right)/\log\{-\frac{\max\left\{R^{2}\right\}}{\left|r_{j}^{1}\right|}\}\\ & =\sum_{j=1}^{J}\alpha \left(G^{1}\right)\times \log(\sqrt{{\left(\mathrm{cap}f_{j}^{1}\right)}^{2}+{\left(\mathrm{mem}f_{j}^{1}\right)}^{2}+{\left(\mathrm{ban}f_{j}^{1}\right)}^{2}})\\ & /\log\{-\frac{\max\{R^{2}\}}{\sqrt{{\left(\mathrm{cap}f_{j}^{1}\right)}^{2}+{\left(\mathrm{mem}f_{j}^{1}\right)}^{2}+{\left(\mathrm{ban}f_{j}^{1}\right)}^{2}}}\} \end{array}$$

The maximum likelihood value is

(A2) $$\frac{1}{{\left(2\pi \right)}^{\frac{J}{2}}{\left(\sigma^{2}\right)}^{\frac{J}{2}}{\left|Ccvs\right|}^{\frac{1}{2}}}\exp\left[\frac{-{\left(l^{G}-1\mu \right)}^{T}Ccvs^{-1}\left(l^{G}-1\mu \right)}{2\sigma^{2}}\right],$$

Taking the logarithm of the above equation,

(A3) $$-\frac{J}{2}\log\left(\sigma^{2}\right)-\frac{1}{2}\log\left(\left|Ccvs\right|\right)-\frac{-{\left(l^{G}-1\mu \right)}^{T}Ccvs^{-1}\left(l^{G}-1\mu \right)}{2\sigma^{2}},$$

The mean $\hat{\mu }$ and variance ${\hat{\sigma }}^{2}$ of the maximum likelihood function can be calculated by the partial derivatives of the mean μ and variance ${\hat{\sigma }}^{2}$ of the above equation:

(A4) $$\hat{\mu }=\frac{1^{T}Ccvs^{-1}l^{G}}{1^{T}Ccvs^{-1}1},$$

(A5) $${\hat{\sigma }}^{2}=\frac{{\left(l^{G}-1\hat{\mu }\right)}^{T}Ccvs^{-1}\left(l^{G}-1\hat{\mu }\right)}{n},$$

The logarithmic maximum likelihood value is

(A6) $$-\frac{J}{2}\log\left({\hat{\sigma }}^{2}\right)-\frac{1}{2}\log\left(\left|Ccvs\right|\right),$$

The covariance matrix of the domain G can be expressed as

(A7) $$\tilde{Ccvs}=\left(\begin{array}{cc} Ccvs & Ccvs^{-1}\\ Ccvs^{-1} & 1 \end{array}\right),$$

$l^{G^{*}}$ is denoted as the target value. The logarithmic maximum likelihood value is

(A8) $$-\frac{J}{2}\log\left(\sigma^{2}\right)-\frac{1}{2}\log\left(\left|Ccvs\right|\right)-\frac{-{\left(l^{G^{*}}-1\hat{\mu }\right)}^{T}{\tilde{Ccvs}}^{-1}\left(l^{G}-1\mu \right)}{2{\hat{\sigma }}^{2}},$$

Make C = ${\left(1-Ccvs^{-1}{}^{T}CcvsCcvs^{-1}\right)}^{T}$. Then, the inverse of the covariance matrix can be expressed as

(A9) $${\tilde{Ccvs}}^{-1}=\left(\begin{array}{cc} Ccvs+CCcvs^{-1} & -CcvsC\\ -CCcvs^{-1} & C \end{array}\right),$$

The expanded likelihood function is

(A10) $$\left[\frac{-1}{2{\hat{\sigma }}^{2}\left(1-Ccvs^{-1}{}^{T}CcvsCcvs^{-1}\right)}\right]{\left(l^{G^{*}}-\hat{\mu }\right)}^{2}+\left[\frac{Ccvs^{-1}Ccvs\left(l^{G}-1\hat{\mu }\right)}{{\hat{\sigma }}^{2}\left(1-Ccvs^{-1}{}^{T}CcvsCcvs^{-1}\right)}\right]\left(l^{G^{*}}-\hat{\mu }\right),$$

The above equation is the quadratic function of $l^{G^{*}}$. The derivative of the above equation, and equal to 0, can be obtained:

(A11) $$\left[\frac{-1}{2{\hat{\sigma }}^{2}\left(1-Ccvs^{-1}{}^{T}CcvsCcvs^{-1}\right)}\right]\left(l^{G^{*}}-\hat{\mu }\right)+\left[\frac{Ccvs^{-1}Ccvs\left(l^{G}-1\hat{\mu }\right)}{{\hat{\sigma }}^{2}\left(1-Ccvs^{-1}{}^{T}CcvsCcvs^{-1}\right)}\right]=0,$$

The solution is

(A12) $$l^{G^{*}}=\hat{\mu }+Ccvs^{-1}{}^{T}Ccvs\left(l^{G}-1\hat{\mu }\right),$$

## Appendix B

There is a set of edges connecting nodes $R=\left\{r_{1},\cdots ,r_{P}\right\}$. The vector sets $r_{1},\cdots ,r_{P}$ are linearly independent.

Make $b_{1}=r_{1}$, then there exists

(A13) $$\|r_{2}^{\prime }\|=\|r_{2}\|\cos\theta =\|r_{2}\|\frac{\left[b_{1},r_{2}\right]}{\left\|b_{1}\|\cdot \|r_{2}\right\|}=\frac{\left[b_{1},r_{2}\right]}{\left\|b_{1}\right\|},$$

then

(A14) $$b_{2}=r_{2}-r_{2}^{\prime }=r_{2}-\|r_{2}^{\prime }\|\frac{r_{2}^{\prime }}{\left\|r_{2}^{\prime }\right\|}=r_{2}-\frac{\left[b_{1},r_{2}\right]}{\left\|b_{1}\right\|}\frac{b_{1}}{\left\|b_{1}\right\|}=r_{2}-\frac{\left[b_{1},r_{2}\right]}{\left[b_{1},b_{1}\right]}b_{1},$$

(A15) $$b_{p}=r_{p}-\frac{\left[b_{1},r_{p}\right]}{\left[b_{1},b_{1}\right]}b_{1}-\frac{\left[b_{2},r_{p}\right]}{\left[b_{2},b_{2}\right]}b_{2}-\cdots -\frac{\left[b_{r-1},r_{p}\right]}{\left[b_{r-1},b_{r-1}\right]}b_{r-1},$$

The orthogonal vector of *R* can be expressed as $v\left(R\right)=\left\{b_{1},\cdots ,b_{P}\right\}$

Then, the extension factor of the domain G can be expressed as

(A16) $$\begin{array}{c} \alpha (G)=[\alpha_{1}(G),\ldots ,\alpha_{p}(G){]}^{T}=0.5T[|p_{d}v_{1}(R)|,\ldots ,|p_{d}v_{p}(R)|]\\ =0.5T(p_{max}-p_{min})[|b_{1}|,\ldots ,|b_{p}|], \end{array}$$
